# Circadian Rhythm of Salivary Immunoglobulin A and Associations with Cortisol as A Stress Biomarker in Captive Asian Elephants (*Elephas maximus*)

**DOI:** 10.3390/ani10010157

**Published:** 2020-01-17

**Authors:** Tithipong Plangsangmas, Janine L. Brown, Chatchote Thitaram, Ayona Silva-Fletcher, Katie L. Edwards, Veerasak Punyapornwithaya, Patcharapa Towiboon, Chaleamchat Somgird

**Affiliations:** 1 Master’s Degree Program in Veterinary Science, Faculty of Veterinary Medicine, Chiang Mai University, Chiang Mai 50100, Thailand; 2 Center of Elephant and Wildlife Research, Faculty of Veterinary Medicine, Chiang Mai University, Chiang Mai 50100, Thailand; 3 Center for Species Survival, Smithsonian Conservation Biology Institute, Front Royal, VA 22630, USA; 4 Department of Companion Animal and Wildlife Clinics, Faculty of Veterinary Medicine, Chiang Mai University, Mae Hia, Chiang Mai 50100, Thailand; 5 Department of Clinical Sciences and Services, The Royal Veterinary College, Hawkshead Lane, Hertfordshire AL9 7TA, UK; 6 North of England Zoological Society, Chester Zoo, Upton-by-Chester, CH2 1LH, UK; 7 Veterinary Public Health and Food Safety Centre for Asia Pacific (VPHCAP), Faculty of Veterinary Medicine, Chiang Mai University, Chiang Mai 50100, Thailand

**Keywords:** Asian elephant, saliva, immunoglobulin A, circadian rhythm, glucocorticoids, welfare

## Abstract

**Simple Summary:**

Salivary immunoglobulin A (sIgA) and cortisol concentrations were measured in Asian elephants to determine circadian rhythm effects and the relationship between both biomarkers. Saliva samples were collected every 4 h from 06:00 to 22:00 h for 3 consecutive days (n = 15 samples/elephant). We used enzyme immunoassays for quantification of sIgA and cortisol concentrations. Both sIgA and cortisol followed a circadian rhythm, although the patterns differed. For both, the highest concentrations were in the early morning hours when elephants began the work day; however, sIgA concentrations were more variable during the day. There was no correlation between the two indices because the pattern of sIgA was quartic, while that of cortisol was linear. We provide basic knowledge for further studies using sIgA as a welfare biomarker.

**Abstract:**

Salivary immunoglobulin A (sIgA) has been proposed as a potential indicator of welfare for various species, including Asian elephants, and may be related to adrenal cortisol responses. This study aimed to distinguish circadian rhythm effects on sIgA in male and female Asian elephants and compare patterns to those of salivary cortisol, information that could potentially have welfare implications. Subjects were captive elephants at an elephant camp in Chiang Mai province, Thailand (n = 5 males, 5 females). Salivette^®^ kits were used to collect saliva from each elephant every 4 h from 06:00 to 22:00 h for 3 consecutive days (n = 15 samples/elephant). Enzyme immunoassays were used to quantify concentrations of IgA and cortisol in unextracted saliva. Circadian rhythm patterns were determined using a generalized least-squares method. Both sIgA and cortisol followed a circadian rhythm, although the patterns differed. sIgA displayed a daily quartic trend, whereas cortisol concentrations demonstrated a decreasing linear trend in concentrations throughout the day. There was no clear relationship between patterns of sIgA and salivary cortisol, implying that mechanisms of control and secretion differ. Results demonstrate for the first time that circadian rhythms affect sIgA, and concentrations follow a daily quartic pattern in Asian elephants, so standardizing time of collection is necessary.

## 1. Introduction

The Asian elephant (*Elephas maximus*) is the official national animal of Thailand, classified as endangered by the International Union for Conservation of Nature (IUCN 2010), and listed in Appendix 1 of the Convention on International Trade in Endangered Species of Wild Fauna and Flora (CITES). Since the logging ban in 1986, thousands of elephants and their mahouts were left without work and took to the streets to beg for food. About a decade later, a new industry emerged for Thai elephants—tourism. In 2017, there were 2673 elephants working in 223 tourism venues throughout the country (National Elephant Institute, Lampang, Thailand). While a few camps offer observation only, most utilize elephants in a variety of scheduled activities, like riding with and without saddles, entertainment shows, and tourist feeding and bathing. Around 900 elephants reside in more than 80 venues in Chiang Mai province. Variation in elephant demographics, work activities, elephant care, and mahout management is evident among the camps of northern Thailand, as recently reviewed by Bansiddhi et al. [1], all of which can affect how individuals cope with the tourist environment.

Assessment of animal welfare relies on measures of physiological function (e.g., health, reproduction, stress) and/or behavior, applied at individual or population levels. The most commonly used biomarkers of stress and, by extension welfare, are glucocorticoids (GC) [2]. In response to an acute stimuli, activation of the hypothalamic-pituitary-adrenal (HPA) axis causes the release of cortisol from the adrenal cortex [3], which then feeds back to inhibit further release to restore homeostasis [4]. In humans and animal models, cortisol is typically measured in blood serum or plasma; however, the potential for inducing stress due to handling and blood collection [5] is a concern for most wildlife species, especially for repeated sampling. Thus, noninvasive approaches that quantify GC metabolites in urine or feces have been developed to assess acute and chronic stress responses in many species [6,7]. Another method—saliva collection—is less invasive than blood and, with a lag time of only 20–30 min, provides almost real time results [7]. Although studies have shown the value of GC for monitoring stress and welfare, including in elephants [8,9], today it is recognized that additional indicators that incorporate multiple physiological systems offer more ways to assess both negative and positive welfare states [10].

Recently, salivary immunoglobulin A (sIgA) has been promoted as a potential biomarker of positive affects [11,12]. Immunoglobulin A is found in many secretory fluids, including saliva and breast milk, and in nasal, gastrointestinal, bronchial, and urogenital secretions [13]. In general, sIgA responds quickly to acute events (positive or negative), increasing or decreasing depending on the stressor. Positive mood inductions related to movies, music, and self-referent statements have been shown to increase sIgA [14], as well as relaxation and massages [13]. However, sIgA concentrations also alter in response to negative effects. For example, depletion of sIgA occurs in humans taking academic exams [15,16], while elevations have been related to mental arithmetic tasks, and reported daily hassles and work demands [13,17]. Studies in mammalian species have linked sIgA to stress as well. In dogs, noise stressors and defense training caused a decrease in sIgA [18,19], while puppies displayed increased sIgA after behavioral testing [19,20]. In pigs, sIgA increased due to restraint stress [21] and isolation [22]. However, before using sIgA as a welfare biomarker, baseline levels must be established, as well as any endogenous patterns. Two studies have measured immunoglobulin A in Asian elephants [23,24], but neither examined specific temporal patterns.

The circadian rhythm is a roughly 24 h cycle in physiological processes, which generally are endogenously generated, although they can be modulated by recurring external cues, such as sunlight, temperature, and sleep-wake and activity cycles [25]. The suprachiasmatic nucleus (SCN) in the hypothalamus serves as the master pacemaker that sets the timing of rhythms by regulating neuronal activity, body temperature, and hormonal signals [26]. Circadian patterns are important to consider when interpreting biological results, to distinguish between basal rhythms and extrinsic effects. It is generally accepted that most mammals exhibit circadian patterns in GC secretion, with concentrations being highest in the morning and lowest at around midnight [16,22,27,28]. Studies in Asian elephants [29,30] have confirmed this pattern in urine [29] and saliva [30,31] samples. IgA also has been shown to have a diurnal pattern. In humans, concentrations decline throughout the day from a morning peak at 08:00–09:00 h. By contrast, in pigs [21] and dogs [18], sIgA concentrations are lowest in the morning (09:00 h), increase during the day (11:00–15:00 h), and then decline at night (17:00 h). There are no reports of circadian rhythms in sIgA in Asian elephants, nor its relationship with the well-studied stress hormone, cortisol. Thus, the goals of this study were to assess temporal patterns of sIgA throughout the day as a potential novel biomarker aiding in the assessment of welfare in elephants, and compare the patterns to those of cortisol. We hypothesized that sIgA in Asian elephants follows a circadian rhythm that is correlated with salivary cortisol.

## 2. Materials and Methods

### 2.1. Animals and Sample Collection

All animal procedures were approved by the Institutional Animal Care and Use Committee, Faculty of Veterinary Medicine, Chiang Mai University, Chiang Mai, Thailand (license number; S2/2561).

Saliva samples were collected from five male and five female Asian elephants aged 34.5 ± 4.7 years (range, 11–54 years) and weighed 3216 ± 306.29 kg (range, 2568–3702 kg) by applying the heart girth equation [32] from an elephant facility in Chiang Mai, Thailand. Samples were collected every 4 h between 06:00 to 22:00 h for 3 consecutive days (n = 15 samples/elephant). Bull elephants were restrained with long chains (30 m) during the day and short chains (5 m) at night. Female elephants were kept unrestrained in a fenced area (1600 m^2^) allowing social interactions during the day (09:00–16:00 h) and on short chains (5 m) at night. Elephants at night were chained inside an open shed with other elephants in close proximity, but with no physical contact. Females participated in tourist feeding and bathing routines twice a day (from 09:00–12:00 and 13:00–16:00 h), while bulls did not interact with tourists. Saliva collection did not interfere with the normal routine of the elephants during the day. At night, lights were turned off at around 22:00 h to allow elephants to rest, so samples were not collected between 22:00 and 06:00 h. Saliva was collected using Salivette^®^ kits (Sarstedt, AG and Co, Numbrecht, Germany) by swiping the absorbent piece inside the buccal area for 30–60 s, which took less than 5 min to complete. Samples were kept in 4 °C coolers for less than 8 h until centrifuged at 1500× *g* for 5 min at 15 °C. Two swabs were collected and the saliva pooled, resulting in an average volume of 500 µL (100–1500 µL) per sample. Saliva was stored at −30 °C until analysis. Samples were analyzed within 3 months as suggested by Ng et al. [33].

### 2.2. Enzyme Immunoassays

#### 2.2.1. Immunoglobulin A

Immunoglobulin A was quantified in Asian elephant saliva by enzyme immunoassay (EIA) as described by Edwards et al. [24] with some modifications. A polyclonal rabbit anti-human IgA antibody (A0262, Dako, Glostrup, Denmark) was diluted to a working concentration of 1 mg/L in phosphate buffered saline (0.01 M phosphate buffer, 0.15 M NaCl, pH 7.2) (PBS) and 100 μL added per well to a 96-well microtiter plate (Nunc-Immuno maxisorp, Thermo Fisher Scientific, Roskilde, Denmark). After incubation overnight at 4 °C, plates were aspirated and washed three times with phosphate buffered saline with tween (PBS-T). Standards (0.39–100 µg/L; I2636, Sigma Aldrich, St. Louis, MO, USA) and saliva samples diluted 1:100 in PBS-T were added in duplicate. Following incubation at room temperature (RT) for 2 h on a plate shaker set to 150 rpm, plates were aspirated and washed three times with PBS-T. A polyclonal rabbit anti-human IgA antibody conjugated to horseradish peroxidase (HRP; P0216, Dako, Glostrup, Denmark) was diluted 1:10,000 in PBS-T and 100 µL added per well before incubation at room temperature (RT) for 1 h on a plate shaker set to 150 rpm. After a final wash step, 100 μL of 3,3′,5,5′-tetramethylbenzidine (TMB) was added per well and incubated in the dark for 10 min at RT. Finally, the reaction was stopped with 50 μL stop solution (1N HCl) and the absorbance measured at 450 nm using a microplate reader (TECAN Sunrise, Salzburg, Austria). Assay sensitivity was 3.37 ng/mL. The EIA was validated for elephant saliva by demonstrating parallelism between serial dilutions of saliva and the IgA standards (*y* = 7.8042*x* + 0.2779, *R*^2^ = 0.986) and significant recovery of IgA added to low concentration saliva before analysis (*y* = 0.935*x* + 0.485, *R*^2^ = 0.997). Samples were analyzed in duplicate; inter-assay coefficient of variation (CV) was 10.49% (n = 3), and the intra-assay CV was 2.36%. 

#### 2.2.2. Cortisol

Concentrations of salivary cortisol were determined using a double-antibody EIA with a polyclonal rabbit anti-cortisol antibody (R4866). Second antibody-coated plates were prepared by adding 150 μL of anti-rabbit IgG (0.01 mg/mL) to each well of a 96-well microtiter plate (Nunc-Immuno maxisorp, Thermo Fisher Scientific, Roskilde, Denmark), and incubated at RT for 15–24 h. The wells were then emptied and blotted dry, followed by adding 250 μL blocking solution (100 mM phosphate, 150 mM sodium chloride, 1% Tween 20, 0.09% sodium azide, 10% sucrose, pH 7.5) and incubating for 15–24 h at RT. After incubation, all wells were emptied, blotted, and dried at RT in a desiccating cabinet (Sanpla Dry Keeper, Sanplatec Corp., Auto A-3, Japan) with loose desiccant in the bottom. After drying (humidity < 20%), plates were heat-sealed in a foil bag with a 1g desiccant packet and stored at 4 °C until use. Neat samples (50 μL) or cortisol standards (50 μL) were added to appropriate wells. Cortisol-horseradish peroxidase (HRP) (25 μL) was immediately added to each well, followed by 25 μL anti-cortisol antibody (except non-specific binding wells) and incubated at RT for 1 h on a plate shaker set to 150 rpm. Plates were then washed four times with wash buffer (1:20 dilution, 20× Wash Buffer Part No. X007; Arbor Assays, Ann Arbor, MI, USA) and 100 μL of TMB dihydrochloride dissolved in phosphate-citrate buffer with sodium perborate (Sigma Aldrich, St. Louis, MO, USA) was added, followed by incubation for 10 min at RT without shaking. The reaction was stopped with 50 μL stop solution (1N HCl) and absorbance was measured at 450 nm by a microplate reader (TECAN Sunrise, Salzburg, Austria). Assay sensitivity based on 90% binding was 0.084 ng/mL. The cortisol EIA was validated for elephant saliva by demonstrating parallelism between serial dilutions of saliva and the cortisol standards (*y* = −10.946*x* + 99.705, *R*^2^ = 0.996) and significant recovery of cortisol added to low concentration saliva before analysis (*y* = 0.7935*x* + 0.0743, *R*^2^ = 0.9987). Samples were analyzed in duplicate; inter-assay and intra assay CVs were 5.48% (n = 4) and 1.46%, respectively.

### 2.3. Statistical Analysis

All analyses were performed using R statistical software version 3.5.1 [34]. Descriptive data were reported as mean ± standard error of the mean (SEM) for both sIgA and cortisol concentrations in each time period, and as overall concentrations for each sex. A generalized least-squares method (GLS) was used to compare differences in sIgA and cortisol concentrations over time. The GLS model was constructed by using nonlinear mixed-effects (nlme) package 3.1-137 [35]. We constructed the model using time period and day of sample collection as the main effects. Individual elephant was defined as a random effect. For GLS modelling, the Akaike information criterion (AIC) was determined from models with different covariance structures, including compound symmetry, autoregressive process of order 1 (AR1), and general correlation matrix with no structure. The compound symmetry structure had the lowest AIC value for the sIgA comparison model and AR1 had the lowest AIC value for cortisol, indicating the best fitted models. Therefore, the structure of the covariance pattern for GLS models for sIgA and cortisol were defined as the compound symmetry and AR1, respectively. Significant differences in mean sIgA and cortisol concentrations between different time periods were analyzed by Tukey’s post-hoc tests followed by examining linear, quadratic, and quartic trend effects over the 24 h cycle using the linear regression model and the locally weighted least squares regression (loess) method. Residuals from the fitted models were tested for normality and homogeneity of variance assumption by plotting standardized residuals versus quantiles of standard normal (QQ normality graph) and plotting standardized residuals versus fitted values, respectively. The plot indicated no violation for both assumptions, thus transformation of the sIgA and cortisol concentration data was not necessary. The scatter plots of sIgA and cortisol values were created using ggplot2 package 3.1.1 [36]. The repeated measures correlation (rmcorr) package 0.3.0 [37] was used to determine the correlation between sIgA and cortisol accounted for inter-individual differences in baseline concentrations. For all statistical tests, the significance level was set at α = 0.05.

## 3. Results

Average sIgA and cortisol concentrations for the three collection periods are summarized in Table 1. The highest average sIgA and cortisol concentrations were observed in samples collected at 06:00 h, while the lowest values occurred between 18:00 and 22:00 h, respectively. Individual concentrations of every time point is provided in Supplementary Materials Table S1.

Average sIgA and cortisol concentrations by sex are summarized in Table 2, with no differences observed at each time point (sIgA: *p* = 0.57, Cortisol: *p* = 0.73).

**Figure 1:**
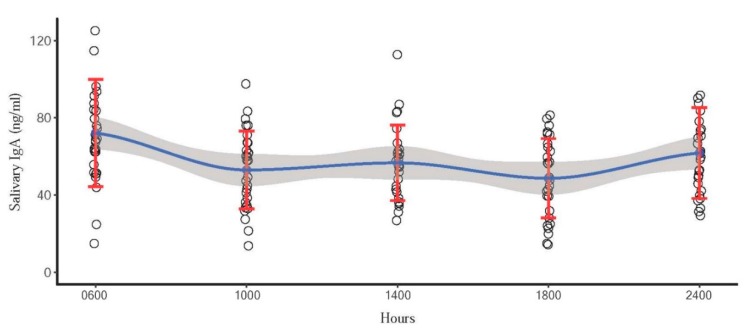
Scatter plot indicating quartic circadian pattern of sIgA concentrations in 10 Asian elephants. Blue dots represent the mean concentration of each time point. Red error bars represent the standard deviation. Blue line represents the trend line, and the shaded area is a 95% confidence interval of the mean.

**Figure 2:**
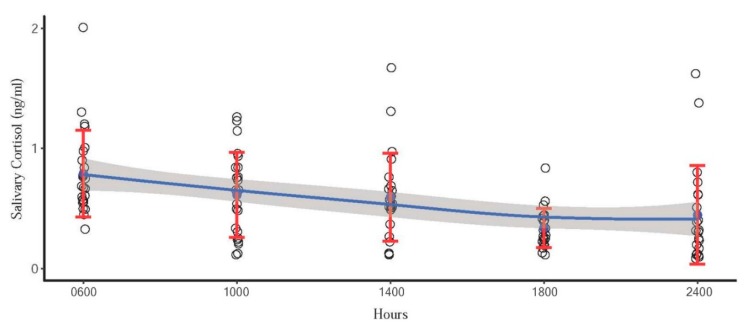
Scatter plot indicating linear circadian pattern of cortisol concentrations in 10 Asian elephants. Blue dots represent the mean concentration of each time point. Red error bars represent the standard deviation. Blue line represents the trend line, and the shaded area is a 95% confidence interval of the mean.

From the GLS model, the effect of time was significant for sIgA (*p* = 0.0001), but only approached significance for cortisol (*p* = 0.06). There was an effect of collection day (sIgA; *p* < 0.0001, cortisol; *p* = 0.0235) for both biomarkers. Even though no effect of time was found for cortisol in the model, post hoc comparisons using the Tukey’s honestly significant difference (HSD) test indicated that mean sIgA concentration at 06:00 h was higher than that at 10:00 h (*p* = 0.002) and 18:00 h (*p* = 0.0001). By contrast, mean cortisol concentration at 06:00 h was only higher than that at 22:00 h (*p* = 0.0373). All data were used to construct a trend line using loess regression analysis. Mean and standard deviation (SD) are presented in Figure 1 and Figure 2 as well as the trend line for IgA and cortisol concentrations. Quartic (*p* = 0.04) trend effects for sIgA (Figure 1), and linear (*p* = 0.002) trend effects for cortisol (Figure 2) were evident throughout the 24 h cycle.

**Figure 3:**
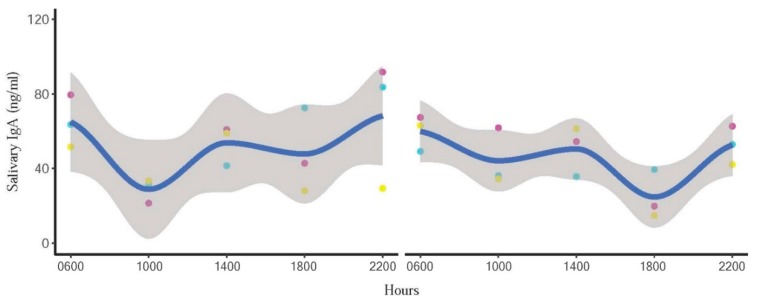
Representative individual profiles showing quartic circadian IgA trends in one male (**left**) and one female (**right**) elephant. Blue line represents the trend line, and the shaded area is a 95% confidence interval. Day 1 = red points, Day 2 = blue points, and Day 3 = yellow points.

**Figure 4:**
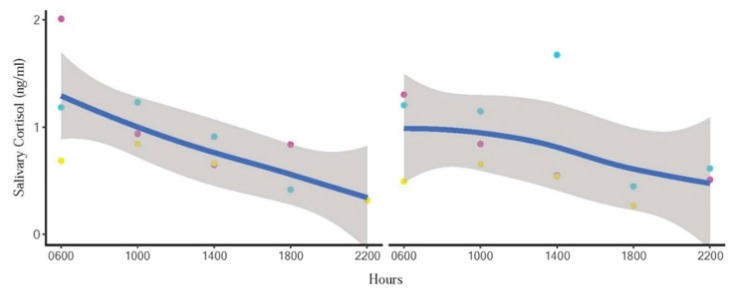
Representative individual profiles showing linear circadian cortisol trends in one male (**left**) and one female (**right**) elephant. Blue line represents the trend line, and the shaded area is a 95% confidence interval. Day 1 = red points, Day 2 = blue points, and Day 3 = yellow points.

**Figure 5:**
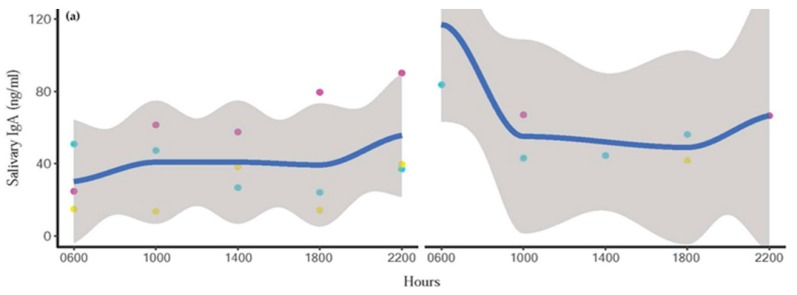

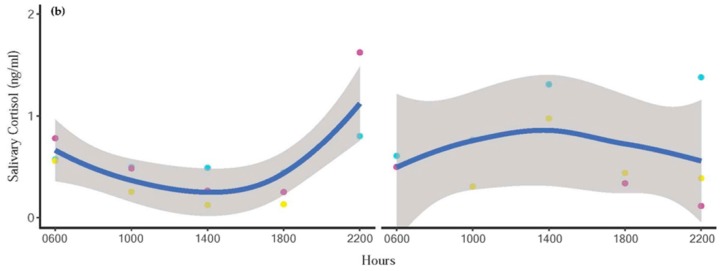
Representative profiles of diurnal (**a**) IgA and (**b**) cortisol trends in one male (left) and one female (right) elephant that did not follow the typical or expected pattern. Blue line represents the trend line, and the shaded area is a 95% confidence interval of the mean. Day 1 = red points, Day 2 = blue points, and Day 3 = yellow points.

sIgA and cortisol patterns of representative individuals depicting quartic and linear effects of the mean are shown in Figure 3 and Figure 4, respectively. However, there was considerable variability and not all elephants followed clear patterns, as indicated in Figure 5. sIgA and cortisol patterns of all individuals is provided in Supplementary Materials Figures S1 and S2. There were missing samples at certain time points because of insufficient volume of saliva for analysis due to a combination of agitated elephants and human error in the collection process (Figure 4 and Figure 5). There was only a weak, non-significant positive correlation (r = 0.099; *p* > 0.05) between sIgA and cortisol.

## 4. Discussion

This is the first study to measure both salivary IgA and salivary cortisol in Asian elephants throughout the day (16-h period), and we found that the trend line from regression analysis followed a diurnal pattern; however, the two were not significantly correlated because cortisol followed a linear pattern, whereas that of sIgA was quartic. The highest concentrations of cortisol were observed at 06:00 h, with the lowest at 22:00 h. By contrast, sIgA concentrations were elevated at 06:00 and 22:00 h, with nadirs at 10:00 and 18:00 h.

Circadian rhythms of IgA are known to vary among species [12]. In humans, sIgA concentrations peak in the morning and decrease throughout the day [38,39]. One study that measured sIgA concentrations over a full 24-h period revealed a gradual increase in sIgA starting at midnight with peak concentrations occurring at 08:00 h the following day [27]. Shirakawa et al. [27] recorded patterns of sIgA in humans coinciding with the sleep-wake time of the subjects, which were between 24:00 and 07:00 h. Our study showed similar peaks in concentrations for sIgA at 06:00 h, which was about an hour after wake time for the elephants. Although the slight increase in sIgA at 14:00 h in our study was not significantly different from 06:00 or 22:00 concentrations, it also was not different from 10:00 and 18:00 h, which agrees with the quartic pattern observed in humans. For elephants in this study, the sleep hours are between 23:00 and 05:00 h. The sIgA increase in samples collected at 22:00 h in elephants corresponded to the time when elephants began their standing sleep period, and agrees with changes associated with the sleep-wake cycle in humans [38]. By contrast, in pigs [21] and dogs [18], peak concentrations are observed during the afternoon. Authors speculate that species-related behavior and differences in daily routines could be the cause of contrasting patterns between species. Like humans, the daily activities of captive elephants are generally fixed, with tourist activities in the morning and afternoon, and a break in the middle of the day. Elephants often sleep in a standing position during these rest periods, which might explain the slight increase in sIgA at 14:00 h. Dogs also show peak concentrations in conjunction with intermittent sleep during afternoon hours [18]. However, the bulls in this study did not interact directly with tourists, yet still showed a quartic pattern, perhaps because although contact was limited, they were still aware of tourist presence. Bull elephants did have a daily routine with mahouts that bathed and fed them at regular intervals, which also could have driven a circadian pattern. Because we were unable to measure a full 24-h cycle, further studies of sIgA concentrations in elephants during sleep would be beneficial to determine the complete cycle and confirm its resemblance to that of humans as compared to other species.

IgA has been measured in multiple sample types (blood, saliva, urine, and feces) across time, including samples from Asian elephants [24]. Samples in the study of Edwards et al. [24] were collected only once a day, with no apparent attention to time, so circadian patterns were not determined. However, concentrations were highly variable, especially for feces and saliva, with serum having the lowest variability. Concentrations in urine were low, with many being undetectable, suggesting it may not be the best sample type to assess this biomarker. In addition to within animal variability, Edwards et al. [24] also found considerable between animal differences, similar to our study. Overall individual mean concentrations ranged from ~7 to 30 ng/mL saliva for five elephants in Edwards et al. [24], and ~41–70 ng/mL for 10 elephants in the present study. Understanding mechanisms driving this significant intra- and inter-animal variability is key to understanding the utility of IgA as a potential health or welfare biomarker.

sIgA concentrations differed somewhat between the present study and that of Edwards et al. [24] in that our overall mean sIgA was more than double in concentration. Possible causes could be related to minor modifications in the assay protocol, which included using lower antibody (1 mg/L versus 10 mg/L) and HRP (1:10,000 versus 1:2500 dilutions) concentrations, although the standard curve range was the same between studies. Climate and daylight hour differences between regions (Washington DC versus Chiang Mai) could have had an effect [40]. For example, Park and Tokura [41] found that brighter light conditions during the day resulted in higher concentrations of sIgA during nocturnal sleep in humans. From Mishra et al. [42], people that travelled from India to Antarctica exhibited increases in sIgA concentrations, which could reflect differences in either climate or day length. No visual differences were evident over six months of longitudinal sIgA data between February and August [24], whereas the Thailand study was conducted in August, so any influence of seasonality may be minimal. Other factors associated with sIgA secretion are age [19,43,44,45,46,47], sex [45], and health status [48,49]. Concentrations of sIgA did not differ between sexes or were related to age in this study, whereas Edwards et al. [24] found the highest sIgA concentrations in the oldest elephant of their study (69 years of age). Another elephant in that study experienced a severe health event indicative of a systemic infection, and showed a four-fold increase in fecal IgA, suggesting it might be a useful health biomarker in elephants [24]. Previous studies in humans have shown decreases in sIgA associated with illnesses, such as upper respiratory tract infections [49] and malignant tumors [48]. Elevated sIgA concentrations also were found after administration of endotoxins to pigs [50] and dairy cows [51], indicating an immune response to pathogens. All elephants in the present study were checked by a veterinarian to ensure there were no underlying health conditions that could interfere with sIgA measures. Although saliva collection generally took less than 3 min, some elephants showed some agitation to the collector’s hand swiping the inside the oral cavity. However, that was not reflected in significantly altered sIgA or cortisol concentrations.

Similar to previous studies, our results indicated a diurnal rhythm for salivary cortisol with peak concentrations in the morning (06:00 h) that gradually decreased throughout the day in a linear trend. Salivary cortisol was highest at 08:00 and lowest at 20:00 h in African elephants [31], which was similar to high values at 07:30 compared to 19:30 h for Asian elephants [30]. This is the same pattern observed for urine, where Brown et al. [29] reported a clear diurnal pattern of glucocorticoid excretion in Asian elephants, with the lowest concentrations observed just before midnight and peak concentrations occurring around 06:00–08:00 hours. However, in this study, clear patterns were not always observed during all collection periods. Five elephants exhibited more random cortisol fluctuations on one or more of the collection days, whereas only three showed the clear diurnal pattern on all three days. Various factors can disrupt normal patterns of cortisol, including stressful events during the day (social disputes, physical accidents, physical restraint) [31,52,53], age, sex, parturition, and environmental factors [52,53,54,55]. Casares et al. [31] revealed that the diurnal salivary cortisol pattern was disrupted by a fight between two zoo African elephants. The incident took place at 14:30 h and ended without human intervention, but the cortisol concentration of both individuals was increased two-fold at 16:00 h during the time it would normally have been declining. In our study, no obvious social disputes occurred between animals; however, the overall daily cortisol concentrations were highest on the first and lowest on the final day of collection, resulting in a statistically significant day effect. This suggests that the sample collection might have induced a mild stress response in some elephants, who then acclimated over time. Figure 2 shows that at 18:00 h, cortisol concentrations present the smallest variation. With low baseline concentrations and variations, sample collection is suggested during this time.

Some studies have reported significant correlations between sIgA and cortisol, including in humans [38] and dogs [56], while others found no such relationships. For example, Escribano et al. [22] revealed no significant correlation between the two parameters from pigs experiencing psychological stress in the form of isolation. Edwards et al. [24] also reported no correlation between salivary IgA and cortisol in Asian elephants from longitudinal samples over a six month period. In this study, no significant correlation between the two indicators was found, as evidenced by a linear downward trend in cortisol throughout the day, while sIgA tended to display a quartic pattern, with concentrations higher in the morning and evening.

## 5. Conclusions

For the first time, circadian effects on sIgA were evaluated in Asian elephants. This study revealed visible daily quartic trends of sIgA, providing basic knowledge of using sIgA as a biomarker for further studies. Results suggest that, just like for cortisol, time of day should be considered for saliva sample collection protocols for monitoring IgA. Moving forward, it will be important to understand differing response mechanisms when using IgA as a welfare indicator—chronic stressors may cause immune suppression and reductions in IgA, whereas acute illnesses could be associated with increases in IgA as part of an immune response to cope with underlying pathology. Thus, interpretation of IgA measures, just like GCs, may not always be straightforward. Both IgA and GCs have been shown to increase in response to acute stressors of a non-immune nature [13,57], and this certainly warrants further investigation before increased IgA concentrations can be considered a positive welfare indicator. As with other potential indicators of well-being, it is also important to understand normal physiological levels both within and between individuals, as well as in response to specific events. Biomarkers must be put into context, preferably by incorporating longitudinal measurements of multiple indicators, including IgA and GCs, to delineate concentrations indicative of an acute immune response or stressor, compared to those associated with longer-term positive or negative welfare states. The methodology described here provides a robust technique to investigate IgA in elephants, and these data provide a necessary baseline to interpret future data alongside other health and well-being measures, to determine whether incorporating IgA measurements will provide useful insight into elephant welfare.

## Figures and Tables

**Table 1:** Daily and overall mean ± standard error of the mean (SEM) concentrations and ranges of salivary immunoglobulin A (sIgA) and cortisol in 10 Asian elephants (n = 5 male, 5 female).

Parameter	Day	Time (hours)					Min-Max
(ng/mL)		06:00	10:00	14:00	18:00	22:00	(ng/mL)
sIgA	1	60.61 ± 3.99	53.40 ± 5.63	43.69 ± 4.00	55.52 ± 6.62	55.04 ± 6.38	22.80–83.70
	2	85.13 ± 11.4	61.62 ± 6.88	71.33 ± 7.27	51.89 ± 6.82	79.13 ± 7.75	19.87–150.18
	3	70.36 ± 8.68	42.85 ± 6.03	58.05 ± 5.4	39.17 ± 6.17	49.17 ± 4.92	13.71–96.19
Overall		72.09 ± 5.07 ^a^	52.96 ± 3.69 ^b^	56.67 ± 3.57 ^a,b^	48.64 ± 3.76 ^b^	61.75 ± 4.29 ^a,b^	13.71–150.18
Cortisol	1	0.82 ± 0.09	0.76 ± 0.10	0.86 ± 0.15	0.71 ± 0.31	0.55 ± 0.15	0.12–3.17
	2	0.87 ± 0.17	0.56 ± 0.14	0.52 ± 0.05	0.34 ± 0.10	0.48 ± 0.18	0.08–2.01
	3	0.63 ± 0.06	0.49 ± 0.14	0.41 ± 0.10	0.28 ± 0.05	0.23 ± 0.06	0.10–1.26
Overall		0.79 ± 0.07 ^a^	0.61 ± 0.07 ^a,b^	0.59 ± 0.08 ^a,b^	0.46 ± 0.12 ^a,b^	0.45 ± 0.09 ^b^	0.08–3.17

^a,b^ Means within rows with different superscripts are significantly different for each biomarker (*p* < 0.05).

**Table 2:** Comparison of overall mean (± SEM) concentrations of salivary immunoglobulin A (sIgA) and cortisol between sexes (n = 5 males, 5 females) throughout three, 24 h periods.

Parameter	Time (hours)					Min-Max
(ng/mL)	06:00	10:00	14:00	18:00	22:00	(ng/mL)
sIgA						
Male	65.88 ± 8.55	52.77 ± 8.34	59.83 ± 7.11	46.06 ± 6.91	60.93 ± 6.45	13.71–125.12
Female	78.74 ± 8.84	53.15 ± 3.58	52.72 ± 4.77	51.62 ± 6.15	62.7 ± 8.7	14.88–150.18
Cortisol						
Male	0.83 ± 0.13	0.69 ± 0.12	0.5 ± 0.08	0.37 ± 0.07	0.42 ± 0.14	0.08–2.01
Female	0.73 ± 0.10	0.52 ± 0.11	0.71 ± 0.15	0.61 ± 0.29	0.44 ± 0.12	0.1–3.17

## Supplementary Materials

The following are available online at https://www.mdpi.com/2076-2615/10/1/157/s1, Figure S1: Individual IgA trends of all elephants, Figure S2: Individual cortisol trends of all elephants, Table S1: Raw data.
