# Supplementary material for: Circadian Rhythm of Salivary Immunoglobulin A and Associations with Cortisol as A Stress Biomarker in Captive Asian Elephants (Elephas maximus)
Source: Animals (Basel). 2020 Jan 17;10(1):157. doi: 10.3390/ani10010157 (PMC7023042; doi:10.3390/ani10010157)
Supplement: Supplementary file 1 [file animals-10-00157-s001.zip › animals-684980.Table S1.docx]

Table S1: Raw data

|  | **Day 1** | | | | | **Day2** | | | | | **Day3** | | | | |
| --- | --- | --- | --- | --- | --- | --- | --- | --- | --- | --- | --- | --- | --- | --- | --- |
| **Elephant/Time** | **6.00** | **10.00** | **14.00** | **18.00** | **22.00** | **6.00** | **10.00** | **14.00** | **18.00** | **22.00** | **6.00** | **10.00** | **14.00** | **18.00** | **22.00** |
| A | 0.57255 | 0.95754 | 0.7618 | 0.32048 | 0.12308 | 0.7654 | 0.73452 | 0.60202 | 0.17035 | 0.16767 | 0.84066 | 1.2627 | 0.36852 | 0.27735 | 0.23458 |
| B | 0.49579 | 0.49019 | 0.44359 | 0.80101 | 0.47079 | 0.7796 | 0.48327 | 0.26554 | 0.25216 | 1.623 | 0.55811 | 0.25408 | 0.12357 | 0.13123 | N/A |
| C | 0.97834 | 0.75655 | 0.52368 | 0.5285 | 0.71875 | 0.59669 | 0.2214 | N/A | N/A | 0.30568 | N/A | 0.11474 | 0.57324 | 0.11372 | N/A |
| D | 0.67394 | 0.65296 | N/A | 0.22535 | 0.24863 | 1.0071 | N/A | 0.51384 | 0.20282 | 0.083837 | 0.44965 | N/A | 0.11371 | 0.33492 | N/A |
| E | 1.1831 | 1.2308 | 0.911 | 0.41697 | 0.31926 | 2.0081 | 0.93684 | 0.64849 | 0.83672 | N/A | 0.68572 | 0.84262 | 0.66224 | 0.56112 | 0.31535 |
| F | 1.203 | 1.1465 | 1.6723 | 0.44629 | 0.61362 | 1.3029 | 0.84176 | 0.54869 | N/A | 0.50896 | 0.4937 | 0.65332 | 0.54065 | 0.26678 | N/A |
| G | 0.66583 | 0.33527 | 0.51133 | 0.39219 | 0.19775 | 0.54869 | N/A | 0.50896 | 0.24742 | 0.40279 | N/A | 0.24062 | 0.22246 | 0.23729 | 0.10787 |
| H | N/A | 0.53864 | N/A | N/A | N/A | N/A | 0.12459 | N/A | N/A | N/A | N/A | N/A | N/A | N/A | N/A |
| I | 0.90117 | N/A | 0.68912 | 0.40313 | N/A | 0.32758 | N/A | N/A | N/A | 0.61798 | 0.74937 | 0.20563 | 0.12756 | 0.19213 | 0.096127 |
| J | 0.60731 | 0.7631 | 1.3091 | 3.1709 | 1.3791 | 0.49823 | N/A | N/A | 0.33737 | 0.11481 | N/A | 0.30614 | 0.97352 | 0.43838 | 0.3873 |

*ng/ml.
