# Supplementary figures and images for: Circadian Rhythm of Salivary Immunoglobulin A and Associations with Cortisol as A Stress Biomarker in Captive Asian Elephants (Elephas maximus)
Source: Animals (Basel). 2020 Jan 17;10(1):157. doi: 10.3390/ani10010157 (PMC7023042; doi:10.3390/ani10010157)

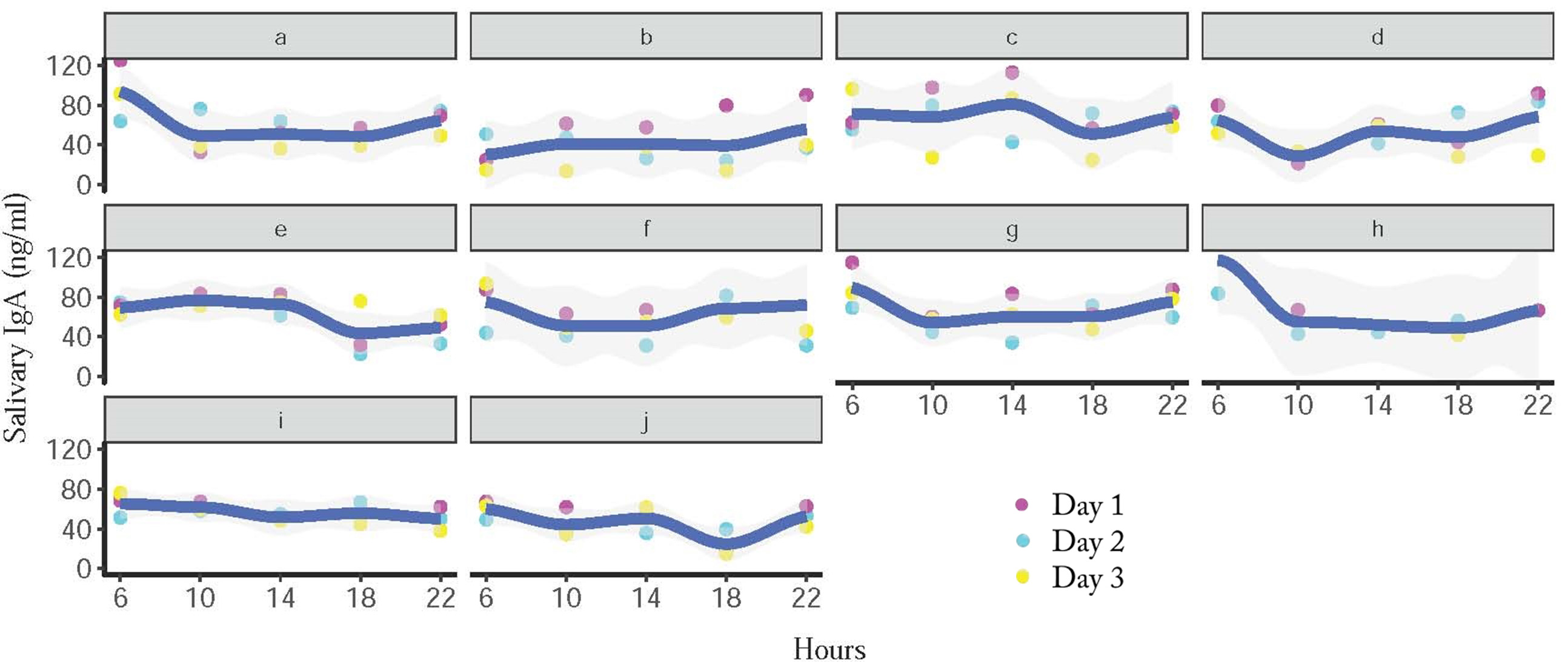

Supplement: Supplementary file 1 [file animals-10-00157-s001.zip › Supplementary 1.jpg]

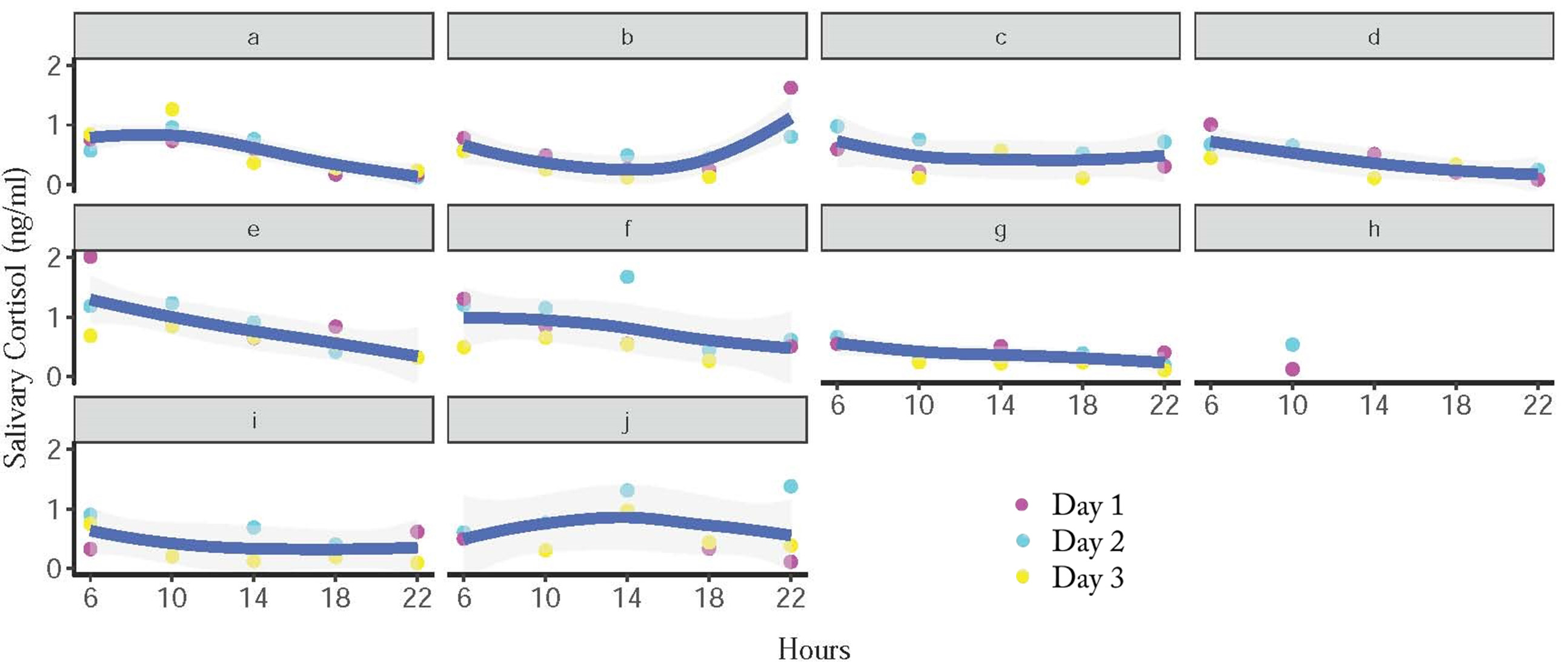

Supplement: Supplementary file 1 [file animals-10-00157-s001.zip › Supplementary 2.jpg]
